# Case Report: Surgical resection combined with chemotherapy for a primary cardiac lymphoma involving right heart structures

**DOI:** 10.3389/fonc.2026.1732411

**Published:** 2026-02-20

**Authors:** Wei Zhu, Xinying Ren, Hanrui Xiong, Man Chen, Boxiang Huang, Bo Peng, Dongqun Lin, Xiaoping Fan

**Affiliations:** 1 Department of Cardiovascular Surgery, Guangdong Provincial Hospital of Chinese Medicine, The Second Affiliated Hospital of Guangzhou University of Chinese Medicine, Guangzhou, China; 2 Department of Radiology, Guangdong Provincial Hospital of Chinese Medicine, The Second Affiliated Hospital of Guangzhou University of Chinese Medicine, Guangzhou, China

**Keywords:** chemotherapy, histopathology, multimodality imaging, primary cardiac lymphoma, surgical treatment

## Abstract

Primary cardiac lymphoma is a highly rare type of malignant tumor that originates from the lymphoid tissue within the heart or pericardium. Surgical treatment is the primary method for obtaining pathological results and relieving obstructions, and chemotherapy is the main method for disease control. We present a case of a 67-year-old woman diagnosed with primary cardiac lymphoma, presenting with shortness of breath and bilateral lower extremity edema after activity. Multimodality imaging results suggest a primary cardiac lymphoma, which was located in the right atrioventricular groove. Surgical resection of the mass was performed and histopathology confirmed the diagnosis of primary cardiac lymphoma. Based on the genetic results, Pola-R-CHP was used for postoperative chemotherapy. Fortunately, no evidence of recurrence of primary cardiac lymphoma was showed 8 months after surgery. Surgical resection combined with chemotherapy has achieved satisfactory results in the treatment of primary cardiac lymphoma.

## Introduction

Primary cardiac lymphoma (PCL) is an exceptionally rare and aggressive extranodal non-Hodgkin lymphoma confined predominantly to the heart and/or pericardium (1). With an estimated autopsy incidence of approximately 0.02%, PCL constitutes only about 1–2% of all primary cardiac tumors and carries a generally poor prognosis (2). Its incidence rate accounts for a mere 1-2% of all primary cardiac tumors (3). Histologically, the majority of cases are of B-cell origin, most commonly diffuse large B-cell lymphoma (DLBCL). Clinical manifestations are highly variable and nonspecific—including dyspnea, arrhythmias, chest pain, or syncope—often leading to delayed diagnosis. In some instances, PCL may precipitate life-threatening complications such as ventricular fibrillation and sudden cardiac arrest.

Owing to its extreme rarity, no standardized treatment protocol has been established, and management often draws from experience with other extranodal lymphomas (4). While systemic chemotherapy remains the cornerstone of treatment, surgical intervention plays a critical role in specific emergent settings—such as significant hemodynamic compromise, cardiac tamponade, or outflow tract obstruction caused by bulky disease (5). Although resection is technically challenging and its effect on long-term survival remains unproven, it can be lifesaving in carefully selected cases.

This report details the clinical course of a patient with PCL involving the right heart structures, who successfully underwent emergency surgical resection followed by adjuvant chemotherapy, illustrating an integrated treatment approach for this challenging disease.

## Case presentation

A 67-year-old female was admitted to our institution with a 10-day history of dyspnea and bilateral lower limb edema. Physical examination revealed no cardiac murmurs or jugular venous distention. Laboratory investigations, including complete blood count, blood chemistry, and cardiac enzymes, were unremarkable apart from an elevated alpha-fetoprotein level of 7.66 ng/mL and an elevated carbohydrate antigen 125 level of 47.2 U/mL. Electrocardiography demonstrated sinus rhythm with T-wave abnormalities.

**Figure 1:**
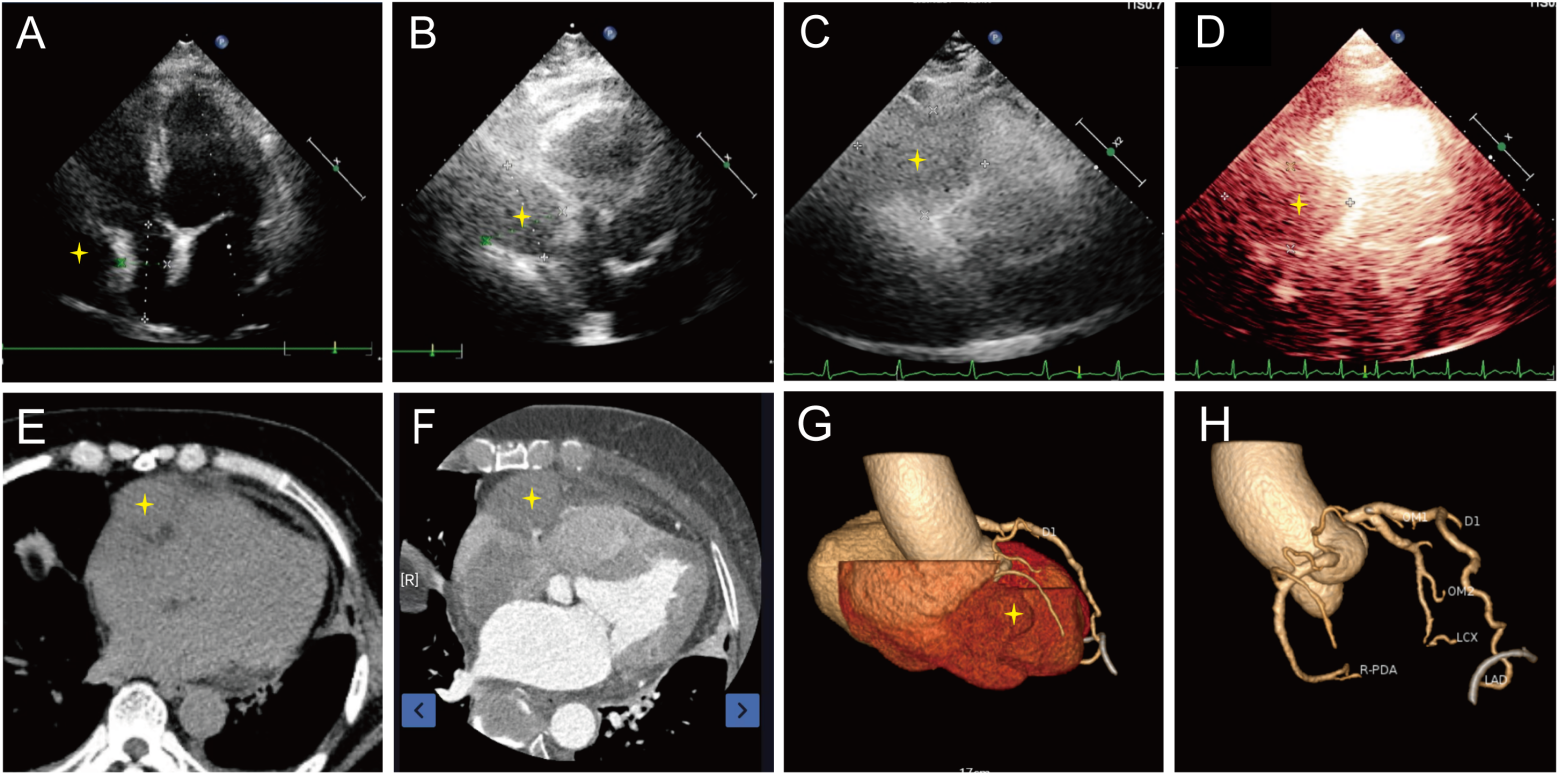
Preoperative imaging. Transthoracic echocardiography revealed a hypoechoic mass in the right atrioventricular groove, with regular contours and ill-defined borders, causing significant compression and deformation of the right atrium **(A, B)**. Myocardial contrast echocardiography showed low perfusion within the mass **(C, D)**. Contrast-enhanced computed tomography characterized the mass in the right atrioventricular groove **(E)** and highlighted its proximity to and compression of the adjacent right coronary artery **(F)**. Three-dimensional reconstruction of the heart **(G)** and coronary arteries **(H)**. Star: Primary cardiac lymphoma.

**Figure 2:**
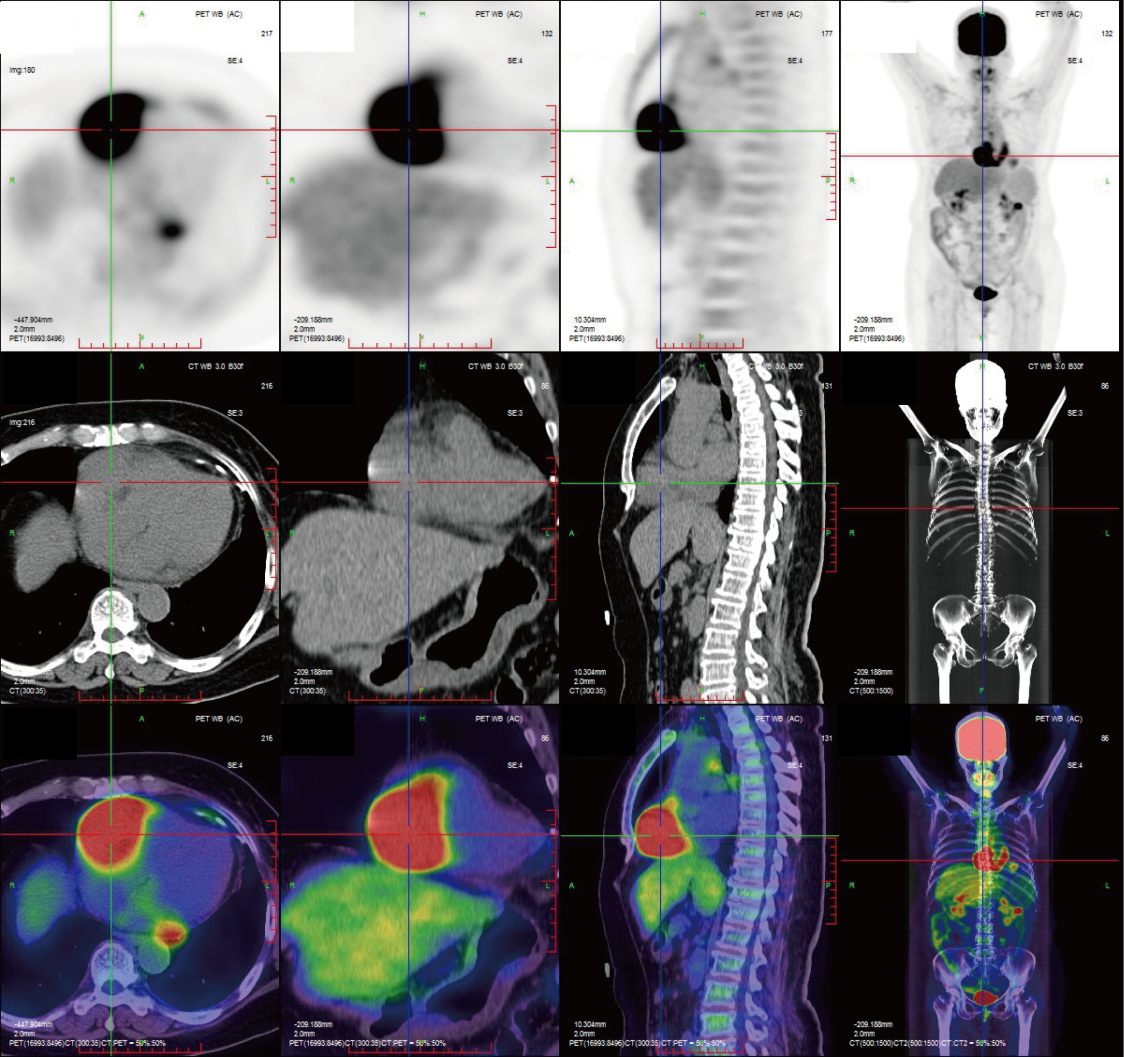
Preoperative PET/CT imaging. PET-CT imaging identified an irregular, iso-dense soft tissue mass in the right atrioventricular region extending toward the left ventricular margin, with markedly increased metabolic activity, highly suggestive of a malignant process. No distant metastatic foci were detected.

Transthoracic echocardiography revealed a hypoechoic mass in the right atrioventricular groove, measuring 58 mm × 48 mm, with regular contours and ill-defined borders, causing significant compression and deformation of the right atrium (**Figures 1A, B**, **Table 1**). Myocardial contrast echocardiography showed low perfusion within the mass, raising suspicion for malignancy (**Figures 1C, D**). Contrast-enhanced computed tomography further characterized the mass in the right atrioventricular groove and highlighted its proximity to and compression of the adjacent right coronary artery (**Figures 1E–H**). Subsequent PET-CT imaging identified an irregular, iso-dense soft tissue mass in the right atrioventricular region extending toward the left ventricular margin, with markedly increased metabolic activity, highly suggestive of a malignant process. No distant metastatic foci were detected (**Figure 2**).

**Table 1:** Dynamic changes in cardiac function parameters.

Parameters	Before surgery	After surgery	Follow up
Left atrium (mm)	41	37	40
Left ventricular end-diastolic (mm)	54	47	49
Left ventricular end-systolic (mm)	29	29	31
Right atrium (mm)	23*46	58*40	34*50
Right ventricular (mm)	24	16	22
Ejection fraction (%)	78	64	65

**Figure 3:**
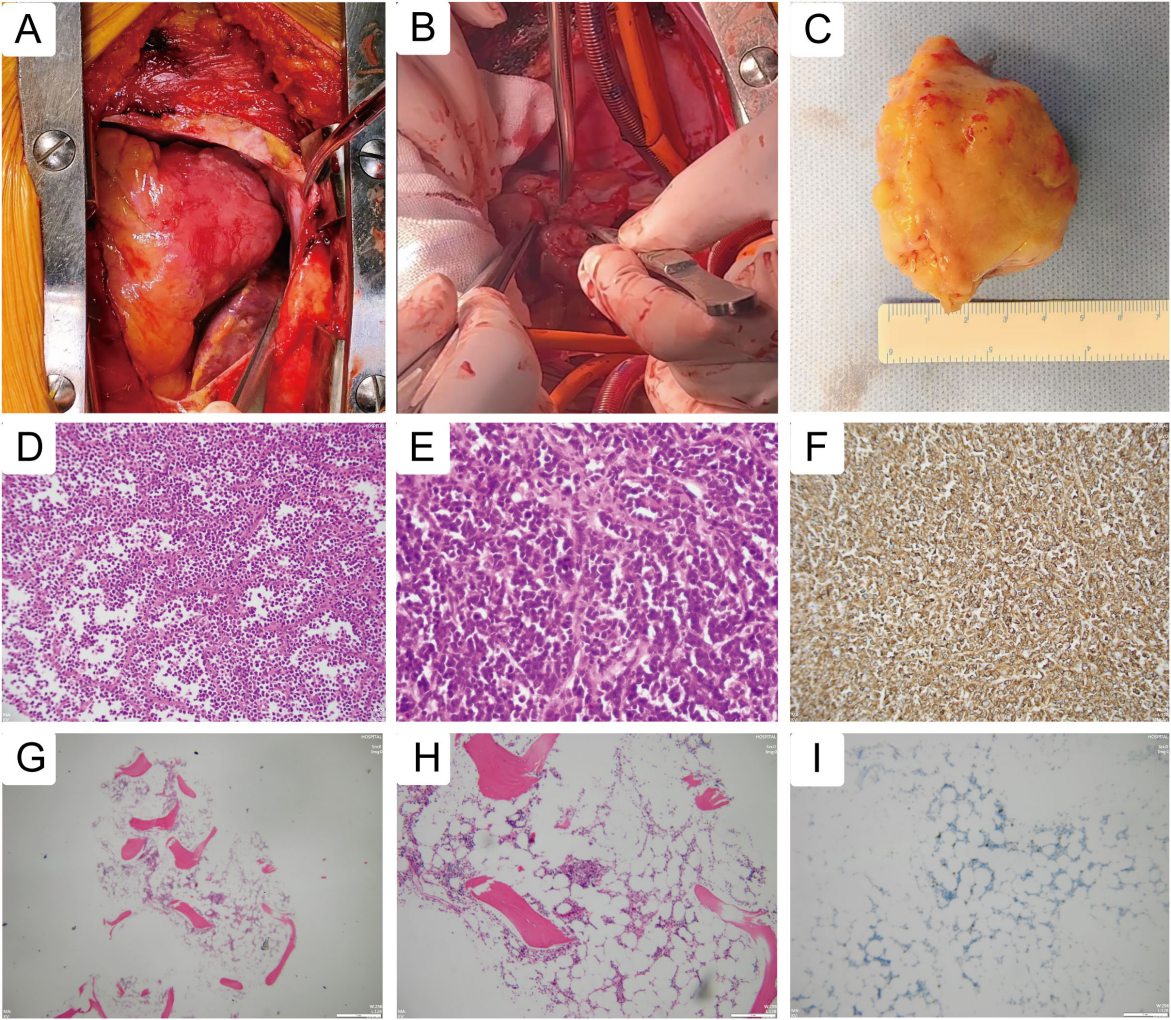
Intraoperative imaging and histopathological examination. **(A)** Intraoperative inspection confirmed the tumor location within the right atrioventricular groove. **(B)** Meticulous dissection was performed along the tumor margins, with particular attention to the interatrial groove and coronary arteries on the right ventricular surface, and the tumor was successfully resected en bloc. **(C)** The resected specimen measured approximately 48 mm × 70 mm × 40 mm. Histopathological examination revealed a diffuse proliferation of medium to large lymphoid cells with irregular nuclei, significant nuclear atypia, and frequent mitotic figures. A starry-sky pattern was observed in some areas, with tumor cells partially arranged perivascularly **(D, E)**. **(F)** Immunohistochemical staining was positive for CD20. Bone marrow biopsy revealed no evidence of lymphomatous involvement, supported by immunohistochemistry showing scattered CD20+ and CD3+ cells without clonal expansion **(G–I)**.

Given the high suspicion of malignancy, the considerable tumor size, and its compressive effects on cardiac structures, a multidisciplinary team recommended surgical resection. Cardiopulmonary bypass was established via cannulation of the ascending aorta and both venae cavae. Intraoperative inspection confirmed the tumor location within the right atrioventricular groove (**Figure 3A**). Meticulous dissection was performed along the tumor margins, with particular attention to the interatrial groove and coronary arteries on the right ventricular surface, and the tumor was successfully resected en bloc (**Figure 3B**). The resected specimen measured approximately 48 mm × 70 mm × 40 mm (**Figure 3C**).

Histopathological examination revealed a diffuse proliferation of medium to large lymphoid cells with irregular nuclei, significant nuclear atypia, and frequent mitotic figures. A starry-sky pattern was observed in some areas, with tumor cells partially arranged perivascularly (**Figures 3D, E**), consistent with an aggressive B-cell lymphoma. Immunohistochemical staining was positive for CD20 (**Figure 3F**), CD79a, MUM1, Bcl-2 (90%), Bcl-6 (90%), P53 (80%), C-myc (40%), CD19 (30%), and focally for CD38 and CD138. Staining was negative for CD3, CD5, CD10, CD23, CD34, CD21, Cyclin D1, and TdT. Molecular analysis confirmed IGH gene rearrangement, with no rearrangements detected in IGK or IGL. Genetic testing identified mutations in BCL7A, BCPR, CD58, H1-4, and PIM1.

The patient’s postoperative course was uneventful, with hemodynamic stability, and she was discharged two weeks later. One month post-discharge, EBV-DNA and CMV-DNA tests were negative. Peripheral blood flow immunophenotyping showed no aberrant populations. Bone marrow biopsy revealed no evidence of lymphomatous involvement, supported by immunohistochemistry showing scattered CD20+ and CD3+ cells without clonal expansion (**Figures 3H, I**).

**Figure 4:**
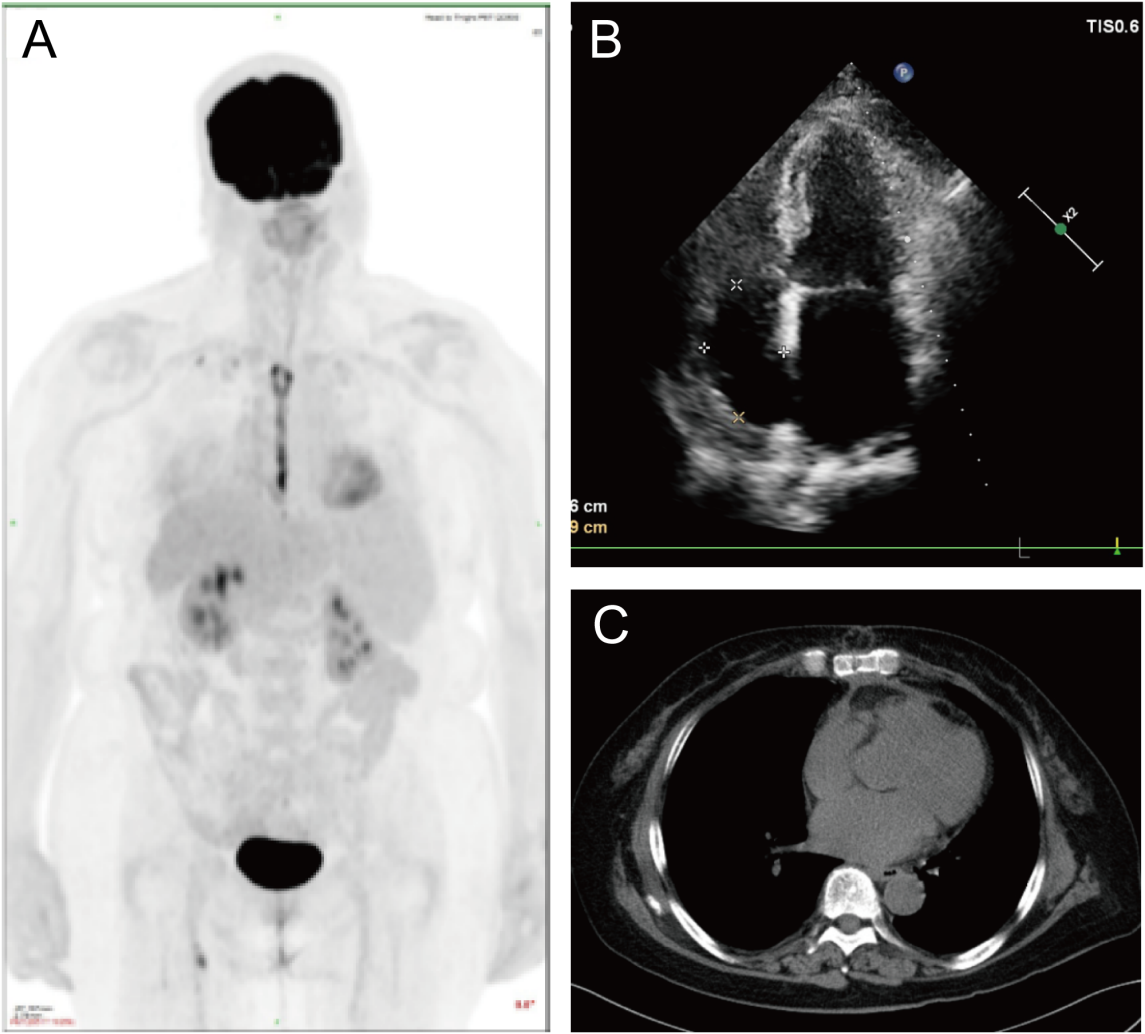
Follow-up imaging. PET-MR **(A)**, echocardiography **(B)**, and chest CT **(C)**, demonstrated no evidence of recurrence or residual disease.

Two months after surgery, the patient received four cycles of Pola+R-CHP chemotherapy(polatuzumab vedotin 90 mg, rituximab 600 mg, cyclophosphamide 1 g, epirubicin 60 mg, dexamethasone 15 mg) (**Supplementary Figure S1**). Treatment was well tolerated, with only mild chemotherapy-induced peripheral neuropathy reported. Follow-up imaging at 8 months post-surgery, including PET-MR (**Figure 4A**), echocardiography (**Figure 4B**), and chest CT (**Figure 4C**), demonstrated no evidence of recurrence or residual disease. The PET-MR scan revealed small patchy areas of increased radioactive uptake in the surgical area, with an SUVmax of 2.63. No other clearly abnormal hypermetabolic lesions were observed throughout the rest of the body. In the upcoming treatment, two more cycles of Pola-R-CHP will still be required.

## Discussion

PCL is an exceptionally rare and aggressive malignancy, accounting for approximately 1% of all primary cardiac tumors and 0.5% of extranodal lymphomas (2, 6, 7). It is predominantly a non-Hodgkin’s lymphoma of B-cell origin, with DLBCL representing the most frequent histological subtype (1, 8). The clinical presentation of PCL is often nonspecific and can be incidentally discovered. The specific symptoms that do manifest are primarily determined by the tumor’s location, size, growth rate, degree of invasion, and tissue friability (9). The right side of the heart, particularly the right atrium, is the most commonly involved site (10), as demonstrated in the present case by the mass originating in the right atrioventricular groove. This ambiguous clinical picture frequently results in diagnostic delays, highlighting the importance of maintaining a high index of suspicion.

Accurate diagnosis necessitates a multimodal imaging strategy. While echocardiography effectively identifies intracardiac masses and assesses their hemodynamic impact, advanced imaging modalities such as cardiac computed tomography and magnetic resonance imaging offer superior anatomical delineation and tissue characterization. PET-CT plays an indispensable role in staging, revealing the tumor’s metabolic activity and confirming the absence of distant disease—a crucial criterion for defining PCL. Nevertheless, histopathological examination remains the definitive diagnostic standard (11). In our patient, the integration of echocardiography, CT, and PET-CT was pivotal in localizing the tumor and informing subsequent therapeutic planning.

Optimal management of PCL requires a multidisciplinary approach. Systemic chemotherapy, particularly the Pola+R-CHP regimen (rituximab, cyclophosphamide, doxorubicin, vincristine, and prednisone), constitutes the cornerstone of treatment and is associated with significantly improved survival outcomes in DLBCL (12). The role of surgery in PCL is primarily diagnostic and palliative. It is indicated both for obtaining tissue for pathological analysis and, critically, for relieving life-threatening mechanical complications such as inflow or outflow tract obstruction and coronary artery compression (13). However, the surgery may result in a temporary decline in postoperative cardiac function, which might be due to the risks associated with extracorporeal circulation, myocardial manipulation, and potential arrhythmias. Therefore, the decision to perform PCL surgery requires a careful risk-benefit analysis. In the current case, surgical resection was imperative to alleviate mass effect on the right atrium and adjacent right coronary artery, thereby averting the imminent risk of hemodynamic collapse and establishing a definitive histopathological diagnosis to guide adjuvant therapy.

Although the operation was performed in the right heart system, the extracorporeal circulation was still used to optimize the surgical strategy, allowing for meticulous dissection in a static environment. Particular attention was paid to resecting the tumor en bloc with as wide a margin as safely possible within the constrained cardiac anatomy, while meticulously preserving the nearby right coronary artery and the atrioventricular groove fat pad to minimize the risk of conduction injury. Intraoperatively, gentle manipulation of the mass was paramount to prevent tumor fragmentation and potential dissemination.

Regarding nervous system prophylaxis, it was not administered in this case. The decision was based on a multidisciplinary tumor board discussion considering that the patient’s lymphoma was confined to the heart without other high-risk features for nervous system relapse at presentation. While the risk in primary cardiac lymphoma is not fully defined, prophylaxis was weighed against potential neurotoxicity, particularly given the planned use of neurotoxic agents like polatuzumab vedotin. The patient will remain under close surveillance for any neurological symptoms.

The prognosis of PCL has historically been poor, with untreated survival frequently measured in months (14). However, the introduction of immunochemotherapy, particularly Pola+R-CHP, has substantially improved clinical outcomes, with several studies reporting complete remission in a significant proportion of patients (15). Achieving long-term survival is possible, underscoring the necessity of rigorous follow-up with serial imaging surveillance, including PET-CT and echocardiography, to detect recurrence. Our patient’s favorable outcome—exhibiting no evidence of disease at an 8-months follow-up after combined surgical debulking and Pola+R-CHOP chemotherapy—further consolidates the critical importance of a timely, integrated treatment strategy tailored to the individual’s clinical presentation for this aggressive malignancy. For this patient, the Pola+R-CHP regimen was selected over standard R-CHOP based on the aggressive histopathological features, the identified high-risk genetic mutations (including PIM1 and BCL7A), and growing clinical data suggesting improved outcomes with polatuzumab vedotin in combination with R-CHP for untreated DLBCL, including those with high-risk features.

Looking beyond first-line therapy, the management of refractory or relapsed PCL follows principles for systemic DLBCL. For patients failing frontline immunochemotherapy, salvage regimens followed by autologous stem cell transplantation remain a standard option for eligible patients (16). Recently, novel immunotherapies have shown remarkable efficacy. Chimeric antigen receptor T-cell (CAR-T) therapy targeting CD19 and bispecific T-cell engagers have demonstrated high response rates in relapsed/refractory DLBCL and represent promising alternatives (17). Furthermore, given the double-expressor phenotype (BCL-2 90%, C-myc 40%) observed in our case, which can be associated with inferior outcomes, targeted approaches are of particular interest. Small molecule inhibitors like the histone deacetylase inhibitor chidamide have shown activity in relapsed/refractory DLBCL and are being explored in maintenance settings (18). While data specific to PCL are lacking, these advanced modalities should be considered in the treatment algorithm for patients with high-risk or relapsed disease.

## Limitations

This study has several limitations inherent to a single-case report. First, the follow-up duration is relatively short for a malignancy with potential for late recurrence. Second, being a report of an individual patient, it cannot establish the generalizability of the treatment approach or definitively isolate the independent contribution of surgical debulking to the overall favorable outcome, which is likely primarily attributable to systemic immunochemotherapy. These limitations underscore the need for larger, multicenter studies to establish optimal management guidelines for PCL.

## Conclusion

Given the aggressive nature and poor prognosis of primary cardiac lymphoma (PCL), timely surgical intervention combined with systemic chemotherapy represents a critical therapeutic strategy. Multimodality imaging is indispensable for accurate diagnosis, preoperative planning, and postoperative surveillance. Furthermore, an integrated treatment approach incorporating targeted immunochemotherapy remains essential for achieving durable remission and improving survival outcomes in patients with this rare malignancy.

## Data Availability

The original contributions presented in the study are included in the article/**Supplementary Material**. Further inquiries can be directed to the corresponding authors.

## Supplementary material

Supplementary Figure 1 Schematic timeline.
