# Supplementary figures and images for: Case Report: Surgical resection combined with chemotherapy for a primary cardiac lymphoma involving right heart structures
Source: Front Oncol. 2026 Feb 20;16:1732411. doi: 10.3389/fonc.2026.1732411 (PMC12962886; doi:10.3389/fonc.2026.1732411)

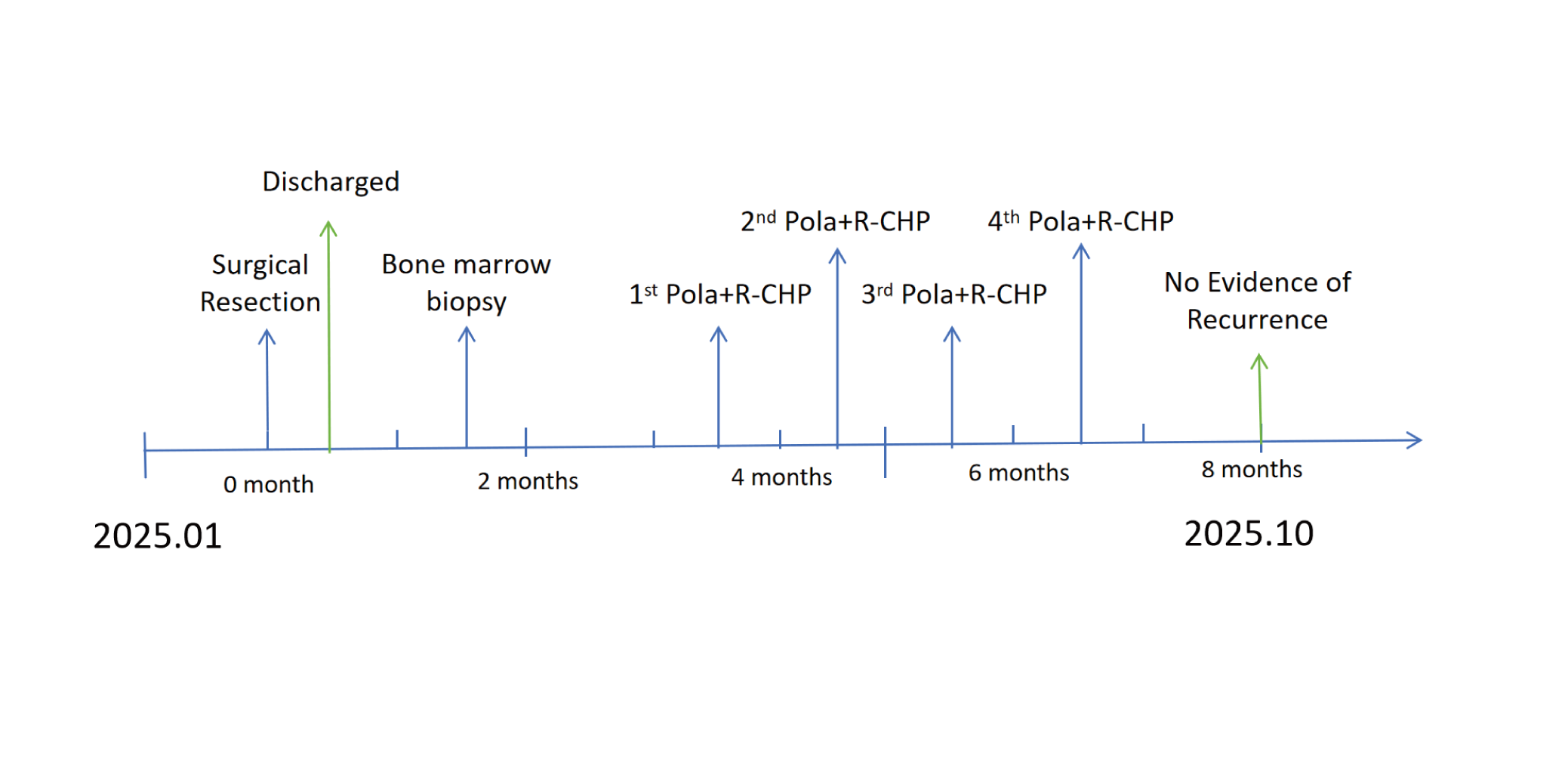

Supplement: Supplementary Figure 1 — Schematic timeline. [file Image1.tif]
